# Supplementary material for: Multi-lingual multi-platform investigations of online trade in jaguar parts
Source: PLoS One. 2023 Jan 23;18(1):e0280039. doi: 10.1371/journal.pone.0280039 (PMC9870105; doi:10.1371/journal.pone.0280039)
Supplement: S1 Table — (DOCX) [file pone.0280039.s001.docx]

S5. Internet penetration, number of internet users and percentage of internet users per country (source: Miniwatts Marketing Group 2020).

| Country | Internet penetration (% total population using internet) |  | Number of internet users | Number of Facebook users | % users per country considering the total of users in Central and South America |
| --- | --- | --- | --- | --- | --- |
| Argentina | 0.931 |  | 41,586,960 | 30,000,000 | 9.9250 |
| Paraguay | 0.896 |  | 6,177,748 | 3,300,000 | 1.4743 |
| Uruguay | 0.882 |  | 3,059,727 | 2,400,000 | 0.7302 |
| Costa Rica | 0.859 |  | 4,296,443 | 3,200,000 | 1.0253 |
| Ecuador | 0.799 |  | 13,476,687 | 10,000,000 | 3.2163 |
| Chile | 0.775 |  | 14,108,392 | 13,000,000 | 3.3670 |
| Brazil | 0.707 |  | 149,057,635 | 139,000,000 | 35.5735 |
| Panama | 0.686 |  | 2,899,892 | 2,000,000 | 0.6920 |
| Peru | 0.676 |  | 22,000,000 | 20,000,000 | 5.2504 |
| Bolivia | 0.675 |  | 7,570,580 | 6,100,000 | 1.8067 |
| Mexico | 0.665 |  | 88,000,000 | 79,000,000 | 21.0017 |
| Colombia | 0.632 |  | 31,275,567 | 29,000,000 | 7.4641 |
| Suriname | 0.598 |  | 340,000 | 310,000 | 0.0811 |
| El Salvador | 0.574 |  | 3,700,000 | 3,400,000 | 0.8830 |
| Venezuela | 0.531 |  | 17,178,743 | 13,000,000 | 4.0998 |
| Belize | 0.513 |  | 200,020 | 200,000 | 0.0477 |
| Guyana | 0.505 |  | 395,007 | 360,000 | 0.0942 |
| Nicaragua | 0.425 |  | 2,700,000 | 2,500,000 | 0.6443 |
| Guatemala | 0.414 |  | 7,268,597 | 6,800,000 | 1.7346 |
| French Guiana | 0.414 |  | 120,000 | 110,000 | 0.0286 |
| Honduras | 0.376 |  | 3,600,000 | 3,400,000 | 0.8591 |
